# Electronic cigarettes have a potential for huge public health benefit

**DOI:** 10.1186/s12916-014-0225-z

**Published:** 2014-12-09

**Authors:** Peter Hajek

**Affiliations:** Wolfson Institute of Preventive Medicine, Barts and The London School of Medicine and Dentistry, Queen Mary University of London, Turner Street, London, E1 2 AD UK

**Keywords:** E-cigarettes, Nicotine, Public health, Controversy

## Abstract

Although there is no doubt that smokers switching to electronic cigarettes (EC) substantially reduce the risk to their health, some tobacco control activists and health organisations discourage smokers from using EC and lobby policy makers to reduce EC use by draconian regulation.

The hostility to EC may be related to a moral belief that nicotine use should be eradicated rather than allowed to morph into a relatively harmless activity. If EC are allowed to compete with cigarettes and develop further, smoking is likely to all but disappear. Discouraging smokers from making the switch and reducing EC competitiveness with cigarettes by unwarranted regulation will delay this opportunity or squander it altogether.

In fact, there is now sufficient evidence available for health professionals to recommend to smokers who cannot stop smoking with existing treatments or do not want to do so, to try several types of e-cigarettes to see if they can find one meeting their needs.

## Introduction

### Electronic cigarettes controversy

Electronic cigarettes (EC) are a consumer product appealing to smokers looking for a safer way to obtain what they want from their cigarettes. From what we know about EC ingredients, toxicology and the chemical and physical processes involved, they can be expected, outside pregnancy, to be at least 95% less harmful than cigarettes [1]. There is now a sufficient body of evidence available on several aspects and effects of EC for recent reviews to conclude that health care professionals and public health bodies should encourage smokers who cannot stop smoking using available treatments, or do not want to do so, to switch to EC [2,3].

Yet at the same time, the World Health Organisation (WHO) have labelled EC a threat to public health, issued a strong advice to smokers not to use them [4], and urges policy makers to limit their use by prohibition or strict regulation [5]. This and other negative campaigns are starting to have an alarming effect of persuading smokers that EC are as harmful as cigarettes [6] and discouraging them from making the switch [7,8].

This commentary argues that EC have a potential to generate substantial public health benefits and that discouraging smokers from using them and regulating EC as severely as cigarettes, or even more severely, is detrimental to public health.

There are manifest humane and logical reasons to encourage smokers who cannot or do not want to stop smoking but want to limit the damage smoking may do to their health to switch to EC. Here is the straightforward case:

There are currently two main products competing for smokers’ custom. One, the conventional cigarette, is responsible for disease and premature death in a substantial proportion of its users. It also continues to recruit new customers from among non-smoking children who try it. The other, EC, is orders of magnitude safer. On current evidence it only appeals to smokers and generates negligible rates of regular use among non-smoking children who try it. Which one would you prefer your nicotine addicted father to use? And if your children were to try a nicotine product, which of these two would you prefer that they lay their hands on?

### Evidence and agendas

For the past few years, scientific journals have been publishing a large volume of commentaries on the EC phenomenon. Most of these focus on hypothetical concerns. Although all commentators now acknowledge that EC are safer than cigarettes, EC are typically presented as a competitor to smoking cessation medications that is possibly less safe and that can somehow increase cigarette use, rather than as a consumer product that competes with cigarettes, and that can make its deadly competitor obsolete.

Some medical organisations which are supposed to protect public health, such as WHO, go further and actively discourage smokers from using EC while lobbying for restricting EC use by regulation. The WHO stance is underpinned by a review they commissioned [9] that has been criticised for an unorthodox use of evidence [10] and illustrates well the anti-EC arguments. Findings that EC vapour contains traces of toxicants is interpreted as a sign of danger and even as a threat to bystanders, even if the levels of these chemicals are well within limits considered safe in the air we breathe [2,11]. Surveys showing that a small proportion of children experiment with EC are presented as a sign of the ‘gateway’ risk despite the fact that virtually no non-smokers progress to daily EC use and that smoking in youth is declining [12]. Where it fits with the negative agenda, trying EC once in the past month is labelled as ‘current use’ which by analogy with ‘current smoking’ is typically interpreted as daily use. Surveys which include only smokers who did not find EC helpful and exclude EC users who stopped smoking are presented as a proof that EC are unhelpful. ‘Dual use’ is presented as a sign of danger despite the fact that it leads to reduced toxin intake [2]. The toxicity of nicotine is exaggerated [13] and the evidence that it makes little if any contribution to smoking related disease and death [14,15] is ignored. Concerns about the twisting of evidence for ideological ends have generated an exchange of letters by large groups of researchers and activists [16-18]. Given the visibility and influence of the activists and medical organisations opposing EC use, there is a risk that these campaigns will discourage or even bar large numbers of smokers around the world from the unquestionable benefits of switching from smoking to vaping. Indeed, alarming signs are emerging that smokers who could benefit from switching to EC now increasingly believe EC are dangerous and they might as well stick to the conventional cigarettes [6-8].

### Why is there a controversy?

EC are a disruptive technology, threatening sales of tobacco products as well as sales of stop smoking medications and so commercially motivated opposition can be expected. The hostility to EC from some tobacco control activists, however, is puzzling. Future textbooks are likely to discuss this phenomenon at length. Here is one hypothesis.

The field of public health is not always rational. Ideology and morality can play at least as big a role as evidence and logic. Public health policies struggle with ideology in areas ranging from abortion to harm reduction strategies in drug addiction and sexually transmitted diseases. One of the possible explanations of the EC controversy is that for some tobacco control activists, any nicotine use is ‘drug abuse’ and abhorrent even if it were to carry no physical health risk. When encountering evidence that EC are much safer than cigarettes, do not attract non-smokers, and promise to reduce smoking-related morbidity, people with this ‘moral stance’ look for objections and counterarguments. Evidence is not needed to discover the truth as the truth is ‘self-evident’ and there is a higher purpose. Evidence is just a tool to gain converts. Nicotine use should be eradicated, not allowed to morph into an activity akin to drinking coffee. An earlier version of the WHO Report to the Framework Convention on Tobacco Control (FCTC), now off-line, betrayed its missionary ethos when it stated that the group’s target is nicotine addiction (that is, nicotine use) ‘independently from its source’ (that is, whether it impacts physical health or not).

Nicotine use, of course, can have negative consequences even if it does not affect physical health. A proportion of users become dependent. However, compared with disease and death caused by combustible non-nicotine chemicals in tobacco smoke, this is a minor consideration. Worries about nicotine use stripped of the health risks of smoking are on par with worries about drinking coffee. Some coffee drinkers do become dependent and spend a fair amount of money and time on their habit, but this does not constitute a major public health issue. It definitely does not justify denying smokers health benefits of stopping smoking just because they would continue to use nicotine and so their conversion to the true virtue would be incomplete.

### How best to appraise the impact of EC?

Negative expectations and concerns can ultimately prove to be correct, even if they were generated by irrational or commercial motives. How should we determine objectively what impact EC are having on public health? For a negative impact, EC use would have to generate an increase in use of cigarettes.

Where commentators worry about gateway effects, undermining tobacco control achievements or re-normalisation of smoking, they should be understood as saying that in their opinion, EC use is generating or is likely to generate an increase in cigarette consumption. When put like this, it appears a highly improbable concern. There is no precedent for a safer technology to increase the use of the less safe competitor. However, hard data on this issue are needed.

Emerging trends are as expected. In the UK where EC are available and taken up by sufficient numbers of smokers, quit rates are increasing and decline in smoking, especially among young people, is accelerating [19-21]. The same is happening in the US [12]. In France and Italy the decline in cigarette sales has been accelerating [22,23]. Such data, of course, cannot determine the cause of these trends. The sales of EC have so far been too low compared to sales of cigarettes for their impact to be clearly visible. More comprehensive studies of the relationship between sales of cigarettes and sales of EC are currently the number one research priority. Comparisons are needed of time trends in sales of cigarettes in countries that allow and that prohibit EC sales, and sales of cigarettes need to be plotted against sales of EC over time. This is needed urgently, before the drastic regulation of EC advocated by the tobacco and pharmaceutical industries and misguided medical organisations stops the effects of EC sales on cigarette sales unfolding and hard data emerging which could provide a rational guidance to policy.

## Conclusions

Today’s e-cigarettes appeal to only a fraction of the smoking population, but if they are allowed to carry on competing with cigarettes as a consumer product and innovate and evolve, there is a good chance that they will continue to improve in offering smokers what they want, cigarette sales will continue to fall, and over the next 10 years, in countries where EC are available and competitively priced, the use of combustible tobacco will virtually disappear. The public health benefit would be huge, even if recreational use of nicotine carries on. If, on the other hand, misleading public health messages discourage smokers from switching and drastic regulations stop EC evolution and make them uncompetitive, the opportunity for a dramatic reduction in smoking related disease and death will be postponed by many years or even missed altogether. Future commentators are likely to consider attempts to remove safer alternatives to cigarettes from the market unethical, however virtuous the missionaries of the nicotine eradication gospel may feel. In the meantime, clinicians facing smokers who cannot or do not want to stop smoking and who follow evidence and common sense rather than ideologically and commercially driven agendas should recommend that their patients try several types of e-cigarettes to see if they can find one meeting their needs.

## Abbreviations

EC: electronic cigarettes
FCTC: Framework Convention on Tobacco Control
WHO: World Health Organisation
